# Fitter frogs from polluted ponds: The complex impacts of human‐altered environments

**DOI:** 10.1111/eva.12751

**Published:** 2019-01-18

**Authors:** Steven P. Brady, Francisco J. Zamora‐Camacho, Fredrik A. A. Eriksson, Debora Goedert, Mar Comas, Ryan Calsbeek

**Affiliations:** ^1^ Biology Department Southern Connecticut State University New Haven Connecticut; ^2^ Department of Biological Sciences Dartmouth College Hanover New Hampshire; ^3^ Museo Nacional de Ciencias Naturales (MNCN‐CSIC) Madrid Spain; ^4^ Estación Biológica de Doñana (EBD‐CSIC) Seville Spain

**Keywords:** amphibian, human‐modified habitats, maladaptation, road salt, roads

## Abstract

Human‐modified habitats rarely yield outcomes that are aligned with conservation ideals. Landscapes that are subdivided by roads are no exception, precipitating negative impacts on populations due to fragmentation, pollution, and road kill. Although many populations in human‐modified habitats show evidence for local adaptation, rarely does environmental change yield outright benefits for populations of conservation interest. Contrary to expectations, we report surprising benefits experienced by amphibian populations breeding and dwelling in proximity to roads. We show that roadside populations of the wood frog*, Rana sylvatica*, exhibit better locomotor performance and higher measures of traits related to fitness compared with frogs from less disturbed environments located further away from roads. These results contrast previous evidence for maladaptation in roadside populations of wood frogs studied elsewhere. Our results indicate that altered habitats might not be unequivocally detrimental and at times might contribute to metapopulation success. While the frequency of such beneficial outcomes remains unknown, their occurrence underscores the complexity of inferring consequences of environmental change.

## 1 INTRODUCTION

Roads are a pervasive feature of the global landscape causing widespread habitat loss and fragmentation (Ibisch et al., 2016; Trombulak & Frissell, 2000). For example, one estimate suggests that 19% of land area in the United States is ecologically affected by roads (Forman, 2000). Roads impact ecosystems through chemical and noise pollution, mortality from road kill, reduced gene flow, and facilitated dispersal of invasive species (Forman & Alexander, 1998; Trombulak & Frissell, 2000). These effects are expected to increase in frequency as our global road network continues to grow, projected to increase 60% in length by 2050 (Dulac, 2013).

Although the ecological influence of roads is well described, evolutionary consequences are poorly understood (Brady & Richardson, 2017). This knowledge gap exists despite the growing appreciation for evolutionary perspectives in other conservation contexts, where insights point to the rapid and complex changes in traits and fitness in human‐modified habitats (Alberti et al., 2017; Carroll, Hendry, Reznick, & Fox, 2007; Carroll et al., 2014). Applying such perspectives to the ecological study of roads—where suites of potentially strong and novel agents of natural selection occur—promises to reshape our understanding of road effects (Brady & Richardson, 2017). For example, a growing number of studies demonstrate changes in behavior, particularly in behaviors important for mate choice and therefore fitness, that are associated with traffic noise (e.g., song production by birds (Slabbekoorn & Peet, 2003), by crickets (Schmidt, Morrison, & Kunc, 2014), and chorusing in frogs (Parris & Schneider, 2009). Moreover, at least one long‐term study has found that traffic can act as a selective agent favoring morphological change, wherein cliff swallows (*Petrochelidon pyrrhonota*) living near roads appear to have evolved shorter wings (associated with more vertical takeoff trajectories) compared to the wider population (Brown & Brown, 2013). These recent insights complement an older literature reporting adaptive responses among plants exposed to road runoff pollution from salt and heavy metals (Briggs, 1972; Kiang, 1982; Wu & Antonovics, 1976).

As a next step toward developing our capacity to understand the role of roads as drivers of evolutionary change, we build on previously reported patterns of road‐mediated population divergence in a pond‐breeding amphibian. Populations of the wood frog (*Rana sylvatica*) breeding in roadside ponds were previously reported to show evidence of maladaptation. Specifically, reciprocal transplant and road salt exposure experiments indicated that larval wood frogs from roadside populations had reduced survival and increased prevalence of malformations compared to experimentally transplanted/exposed woodland populations (Brady, 2013, 2017).

In the present study, we set out to expand on these previous results by investigating whether larval wood frogs in a new set of study populations would show similar patterns of maladaptation. In addition, we tested whether this hypothesized pattern of maladaptation would be present in adulthood. We tested for differences between roadside and woodland populations in several ways. First, we measured morphological and locomotor performance variables that correlate with fitness components in wood frogs. Second, we directly measured three components of fitness (larval survival, adult survival, and fecundity). Third, we assessed variation in age structure in adults as a demographic signature of differences between demes. In contrast to previous studies, we found that roadside wood frogs in these new study populations have higher measures of fitness and indeed in many cases outperform frogs from more pristine environments. We discuss our results in terms of the implications for assessing evolutionary outcomes in human‐modified habitats.

## 2 METHODS

### 2.1 Natural History, study site, and adult frog collection

The wood frog, *R. sylvatica,* is a medium‐size anuran (45–60 mm snout–vent length—SVL—in our study system) that inhabits forests in vast regions of North America. It is the only amphibian to live north of the Arctic Circle. Wood frogs in our study population have a highly synchronized breeding period that follows the onset of above‐freezing temperatures and spring rains, occurring typically in April. Adult wood frogs typically breed in vernal or other ephemeral ponds (hereafter “pools”), where most individuals arrive within a few days of each other. Females lay egg masses typically containing 700–1,000 eggs, which are usually fertilized by a single amplexed male. Eggs hatch into larvae (tadpoles) after approximately 15–20 days and then, approximately 60–70 days later, metamorphose into froglets, and disperse from their natal pool into terrestrial habitat. Larval mortality and juvenile mortality are high, and wood frog tadpoles are consumed mostly by aquatic invertebrates (Groner et al., 2013; Relyea, 2002) whereas newly metamorphosed individuals are consumed primarily by snakes (Arnold & Wassersug, 1978).

We studied nine replicate populations of wood frog near Norwich VT (43.7153°N, 72.3079°W) in spring 2014. Populations were characterized as one of two types: “woodland” populations (*N* = 5) originated from pools located at least 150 m away from the nearest road and surrounded by forested habitat while “roadside” populations (*N* = 4) were road‐adjacent, located within 10 m of paved roads. Woodland pools were all separated from roadside pools by at least 500 m. We measured specific conductance (a reliable surrogate for road salt contamination in this system), pH, and dissolved oxygen in these pools on 7 May 2015 using a YSI‐water quality monitor (YSI Inc., Yellow Springs, OH, USA). We recorded average weekly water temperature (recorded at 1‐hr intervals) using iButton data loggers deployed at 10 cm depth. These data were all approximately normally distributed, and we analyzed differences between pool types using ANOVA. All analyses (here and throughout) were conducted using JMP v. 12 statistical software. In any cases where interactions were evaluated, only significant interactions were retained for inference. In cases with nonsignificant interactions, the interaction term was dropped from the model and main effects were evaluated instead.

To capture incoming adult frogs, we erected drift fences around these pools. To construct drift fences, we buried three to five 10 L plastic buckets at intervals of 5–10 m along pool boundaries and staked one‐meter‐high fences of 2 mm mesh size hardware cloth as drift‐fence barrier. Buckets were checked daily. Adult frogs captured from drift fences were held in buckets and brought back to the laboratory where they were paired to amplex with an opposite‐sex partner from the same breeding pool chosen at random. Each pair was placed in its own 1 L plastic aquarium with 500 ml of dechlorinated tap water, one leaf (*Quercus *spp.), and one small stick onto which females could attach their eggs during oviposition. Following oviposition, adult frogs were removed and transferred to individual aquaria to prevent egg mass disruption. During their stay in the laboratory, we kept frogs in a temperature‐controlled room, at 11°C with a 14:10 light–dark cycle. No frog suffered any damage as a consequence of this study. Most individuals were returned to their original pool immediately after experimental procedures. The one exception was a subset of adult frogs used to estimate survival selection in terrestrial enclosures (below). These were chosen such that we held equal numbers of each sex from both woodland and roadside populations, but were otherwise selected at random.

### 2.2 Morphology and jumping performance

As a measure of body size, we recorded body mass (nearest 0.1 g), using a digital balance. Females were measured twice, first before and then again 24 hr following oviposition. We used a digital caliper to measure the following morphological traits for each individual: forearm length (mm), tibiofibular length (mm), and number of nuptial pads (of male frogs only). We measured nuptial pads because swollen nuptial pads are used by males to secure themselves in amplexus with females and likely influence male reproductive success (Greene & Funk, 2009).

We measured jumping performance of male and female frogs. Female frogs were jumped during two separate trial periods, once prior to oviposition and a second trial 24 hr after oviposition. Male frogs were jumped only once (after breeding). For each trial, we removed an individual frog from our temperature‐controlled room and immediately placed it on a mat of brown paper. The jumping arena was 1 m wide and 4 m long, with vertical barriers on both sides that prevented escape during jumping trials. Frogs were motivated to jump forwards by gently touching their hindquarters. We recorded up to five consecutive jumping distances for each individual, marking their progress on the arena floor with a black permanent marker. Only “good” trials, in which the frog made forward progress without deviating to the side of the arena or attempting to evade the researcher, were recorded. We recorded the average and maximum jumping distance for each individual. Following completion of jumping trials, we collected a toe‐clip from each individual to be used for subsequent skeletochronology analyses to estimate age (see below). All individuals were clipped at the third toe of the right hind limb except for those individuals used in our adult survival experiments, which were clipped at one toe on each foot in unique combinations for individual identification. Toe‐clipping has been shown to have little or no effect on adult amphibian locomotion and survival (Ginnan, Lawrence, Russell, Eggett, & Hatch, 2014; Zamora‐Camacho, 2018).

Morphological and jumping distance metrics were all approximately normally distributed. We used analyses of variance (ANOVA) to test whether each of these variables differed between population type, sex, and their interaction. We included hind limb length as a covariate in analyses of jumping performance and age as a covariate in analyses of size.

### 2.3 Fitness components

#### 2.3.1 Fecundity

Following the assignment of eggs into the reciprocal transplant experiment (described below), we photographed egg masses (Brady, 2013) to count total egg numbers laid by each individual. We used analyses of variance (ANOVA) to test the hypothesis that fecundity varies between population types. Female body mass was included as a covariate in analyses of fecundity.

#### 2.3.2 Larval survival via reciprocal transplant experiment

We collected egg masses laid by females in the laboratory (see above) and used a 5× dissecting microscope to verify that eggs were fertile and actively undergoing cell division before splitting two groups from each egg mass, targeting approximately 60 eggs per group (mean ± *SE* = 66.4 ± 1.2). For each egg mass, we haphazardly assigned one group to its natal pool and the other to its paired pool of opposite type (i.e., roadside and woodland). Pool pairs were assigned to maximize geographic proximity and approximate likeness in surface area and canopy cover. Each group of eggs was randomly assigned either to its maternal (roadside or woodland) environment or to the alternative pool type. Eggs were held in 500 ml plastic containers placed on ice during transport to experimental pools, at which time they were transferred into 15 L floating cages and introduced to the pool. Cages were constructed of plastic boxes with mesh‐covered openings that allowed free passage of pool water, but excluded predators. Exterior surfaces of boxes were equipped with foam floats such that boxes were suspended just below the water line of experimental pools and eggs were maintained at depth comparable to naturally laid clutches. We monitored egg cages twice per week and terminated the experiment when all eggs had either hatched or else died. In total across all pools, we sampled 100 egg masses from amplexed pairs in the laboratory and stocked 13,205 embryos into reciprocal transplant cages. The proportion of surviving embryos in our transplant experiments had a Poisson distribution. We used a generalized linear model to test whether survival differed across the interaction of population type and environment.

#### 2.3.3 Adult survival experiment

Adult frogs that were measured and scored for performance metrics (above) were individually toe‐clipped for permanent identification (without clipping an extra toe for skeletochronological analysis). We released fifty frogs into each enclosure (approximately equal sex ratio and equal numbers of frogs from woodland and roadside populations to each enclosure). Enclosures were constructed from the same 2 mm hardware cloth used to construct drift fences, and the bottom 10 cm of each mesh wall was buried in the ground. The two enclosures were constructed next to each other such that they shared a common wall, and were installed 25 m away from one of our woodland study pools under the forest canopy. Although the walls of each enclosure were sufficiently high (1 m) to ensure that no frog could escape, enclosures were left open from above to allow some natural predation by birds and/or small mammals.

Each enclosure measured 20 m^2^ and contained mixed leaf litter (*Quercus *and *Acer *spp.). We did not modify the substrate in any way, except to move fallen logs and debris that otherwise impeded the fence. We also verified that no other vertebrates (e.g., rodents, salamanders) were present in the enclosures at the start of our experiment. We supplemented the natural arthropod communities with a one‐time release of ~100 *Achaeta* crickets into each enclosure at the time of frog introduction. We performed an informal survey of frog density after 1 week to gauge mortality rates so that we knew when to terminate the experiment. After 2 weeks, we surveyed surviving frogs exhaustively by carefully raking the leaf litter into small piles, then manually sorting through the leaves and removing surviving frogs. Sorted leaf litter was removed by hand from each enclosure until both enclosures were raked to bare ground. We also removed logs and other debris to ensure that all surviving frogs were recaptured.

Survival data have a binomial distribution (live/die), and we tested for differences in adult survivorship in our two experimental enclosures using generalized linear models with logit link functions.

#### 2.3.4 Age structure

To test the hypothesis that roadside and woodland populations have different adult age structure, we estimated the numbers of individuals in different age classes at each pool type using a skeletochronological analysis. Ectotherms with indeterminate growth that exhibit cyclical periods of growth and resting produce lines of arrested growth (LAGs) in skeletal tissue that were used to estimate age (in our study system, each LAG corresponds to one year because there is only one period of resting per year: hibernation). For this study, we collected the third toe of the right hind limb to estimate age using phalanx skeletochronology (Comas, Reguera, Zamora‐Camacho, Salvadó, & Moreno‐Rueda, 2016). Prior to mounting samples, glass slides used for skeletochronology were prepared by submerging for at least 5 min in a solution of glycerol (5 g/L) and chromium (III) potassium sulfate (0.5 g/L). Slides were then oven dried for 24 hr and refrigerated until use. We conducted several trials to estimate optimal duration of decalcification. Finally, samples were decalcified in 3% nitric acid for 4 hr. Decalcified samples were preserved in phosphate‐buffered saline (PBS) solution with sucrose for at least 48 hr at 4ºC and embedded in gel OCT (optimum cutting temperature). Embedded samples were cross‐sectioned at 10–12 µm with a freezing microtome (Leica) at University of Barcelona. Cross sections were recovered onto slides and stained with Harris hematoxylin by submersion for 20 min. Slides were washed in tap water for 5 min to rinse excess stain prior to dehydration in an alcohol series.

Cross sections were examined for the presence of LAGs using a light microscope (Zeiss) at magnifications from 40 to 125×. We took several photographs using a camera (Axiocam105 Zeiss) of various representative cross sections, discarding those in which cuts were unsuitable for examining the LAGs. We selected diaphysis sections in which the size of the medullar cavity was at its minimum and that of the periosteal bone at its maximum (Comas et al., 2016). The number of LAGs detected in the periosteal bone was independently counted three times by the same person but on different occasions, always blindly regarding specimen identification (Comas et al., 2016). Frogs were collected in early spring, after hibernation. Consequently, the outer edge of the bone was not discernible from the last LAGs deposited during hibernation. For that reason, it was counted as a LAG.

Age was summarized by the number of individuals from each sex in each of four age classes (2‐, 3‐, 4‐, and 5‐year‐olds). We used chi‐square tests to evaluate whether age distribution differed with respect to population type and sex.

## 3 RESULTS

Compared to woodland pools, roadside pools had significantly higher specific conductance (ANOVA *F*_1,7_ = 58.55, *p* < 0.0001), higher water temperatures (ANOVA *F*_1,7_ = 15.67, *p* = 0.006), and higher levels of dissolved oxygen (ANOVA *F*_1,7_ = 13.04, *p* = 0.01). Roadside and woodland pools did not differ in pH (ANOVA *F*_1,7_ = 0.15, *p* = 0.71) or in their maximum depth (ANOVA F_1,7_ = 3.73, *p* = 0.09). In roadside pools, specific conductance ranged (top of water column/bottom of water column) from 300/800 µS to 400/3,200 µS. On average, roadside pools were 21–66 times more conductive (top – bottom), 78% warmer, and had 67% more dissolved oxygen than woodland pools (Table 1). In addition to the presence of road salt as indicated by the elevated specific conductance, roadside pools were visibly more polluted than woodland pools, with the presence of refuse (e.g., aluminum cans, discarded paper waste) noted at all roadside pools but never at woodland sites.

**Table 1:** Environmental differences between population types revealed that roadside pools had higher levels of dissolved oxygen, had higher conductivity (salinity), and were warmer compared to woodland pools. Table shows mean values ±(1*SE*). Specific conductance was measured at the top and bottom of the water column in roadside because road salt runoff causes a halocline across the water column

Environmental variable	Population type		*P*‐value
	Roadside	Woodland	
Dissolved O_2_ (mg/L)	9.07 (0.75)	5.42 (0.67)	0.009
pH	7.08 (0.29)	6.93 (0.26)	0.711
Conductivity top (µS)	511 (26)	24 (22)	<0.001
Conductivity bottom (µS)	1,674 (626)	–	0.015
Depth (cm)	78 (11)	49 (9.8)	0.094
Temperature (°C)	13.62 (1.13)	7.62 (1.01)	0.005

### 3.1 Morphology and jumping performance

**Figure 1:**
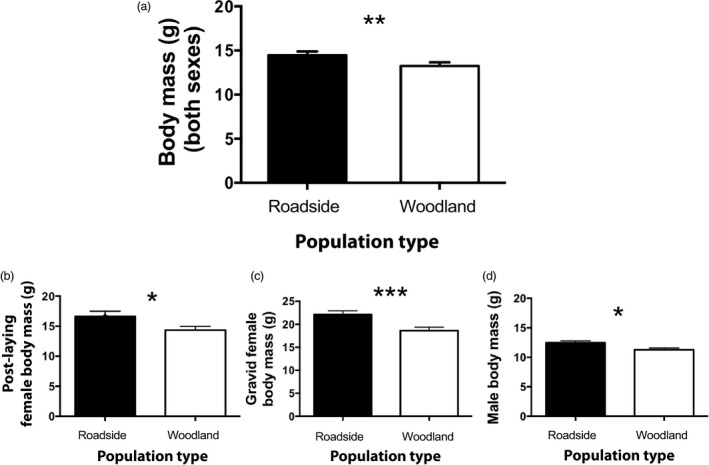
Frogs from roadside pools (filled bars) differed in body size (i.e., mass) compared to frogs from woodland pools (open bars) (a). In particular, female body mass was greater in road size pools both after oviposition (b) and even more so while gravid (c). Male body size was also larger in roadside pools (d) although the difference was smaller compared with females. Bars show least square mean values (±*SE*) after accounting for variation due to age. *p < 0.05, **p < 0.01, ***p < 0.001

We captured a total of 200 adult frogs from drift fences (31 females and 71 males from woodland pools, 36 females and 62 males from roadside pools). The following analyses of body size include only the subset of frogs to which we could assign an age category (*N* = 156), but results were qualitatively identical when we analyzed the full dataset without the age covariate. Across sexes, frogs from roadside pools had greater body mass than frogs from woodland pools (ANOVA *F*_1,133_ = 9.10, *p* = 0.003; effect of age *p* < 0.001; Figure 1a). This difference in body mass was larger for females than males. Post‐laying females from roadside pools were 16% larger than post‐laying females from woodland pools (16.62 ± 0.85 g vs. 14.33 ± 0.65 g, respectively; ANOVA *F*_1,29_ = 5.74, *p* = 0.02; effect of age *p* = 0.22; Figure 1b). Differences were qualitatively similar but greater in magnitude when we compared gravid females from roadside pools (22.11 ± 0.71 g) and woodland pools (18.59 ± 0.78 g), representing a 19% increase in average body mass in roadside gravid females (ANOVA *F*_1,60_ = 11.15, *p* = 0.001; Figure 1c). We further explore this difference in reproductive investment below. Males from roadside pools were about 11% larger than males from woodland pools (12.48 ± 0.29 g vs. 11.27 ± 0.31 g for roadside and woodland populations, respectively; ANOVA *F*_1,96_ = 10.38, *p* = 0.001; effect of age *p* = 0.51; Figure 1d).

The nuptial pads of male frogs were more abundant on individuals from roadside populations (2.83 ± 0.07 mm) compared to woodland populations (2.63 ± 0.07 mm), a difference that was nearly significant after accounting for variation due to forearm length (ANOVA *F*_1,129_ = 3.73, *p* = 0.06; effect of limb length *p* = 0.0001; Figure 3c).

**Figure 2:**
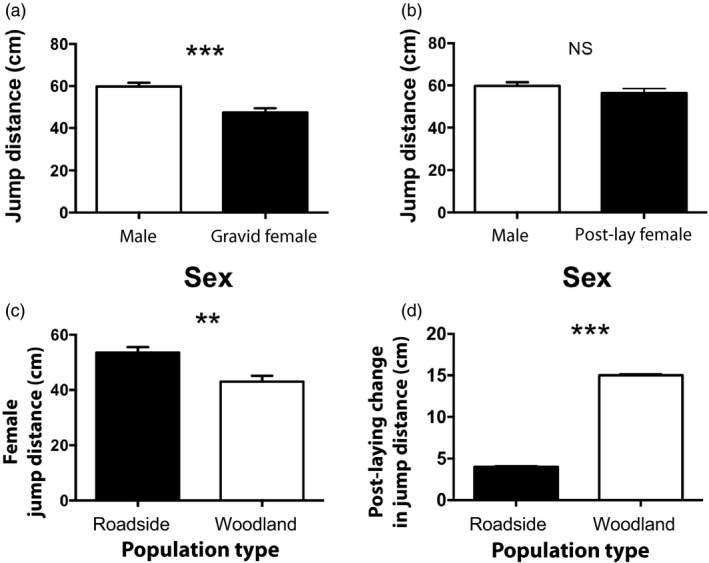
Maximum jumping distance varied both between the sexes (top panels) and between population types (lower panels). In particular, males jumped significantly further compared to gravid females (a). This difference disappeared post‐oviposition (b). Gravid females from roadside pools jumped further than gravid females from woodland pools (c) and experienced a significantly lower cost of carrying eggs as evidence by the smaller change in jumping performance pre‐ and post‐laying (d). Bars show least square mean values (±*SE*) after accounting for variation due to hind limb length. **p < 0.01, ***p < 0.001

Males from roadside and woodland pools did not differ in their maximum jumping distance (ANOVA *F*_1,88_ = 0.37, *p* = 0.54; effect of limb length *p* = 0.14). Males exhibited larger maximum jumping distances compared to gravid females (59.81 ± 1.67 cm vs. 47.50 ± 2.02 cm for males and females, respectively; ANOVA *F*_1,141_ = 16.28, *p* < 0.001; effect of limb length *p* = 0.35; Figure 2a) but not when compared to post‐laying females (59.81 ± 1.67 cm vs. 56.46 ± 2.18 cm for males and females, respectively; ANOVA *F*_1,134_ = 1.03, *p* = 0.31; effect of limb length *p* = 0.46; Figure 2b). Despite carrying a larger mass of eggs and a greater total number of eggs, gravid females from roadside pools exhibited larger maximum jumping distances than gravid females from woodland pools after accounting for variation due to hind limb length (53.59 ± 1.97 cm vs. 43.0 ± 2.14 cm; ANOVA *F*_1,141_ = 7.58, *p* = 0.007; effect of limb length *p* = 0.76; Figure 2c). Moreover, the change in jumping performance before and after egg‐laying was smaller for roadside females than for woodland females. Roadside females jumped four fewer centimeters when gravid than they did after laying (51 ± 2.21 vs. 55 ± 2.34 cm) whereas woodland females experienced a 16 cm increase in jumping distance after oviposition (43 ± 2.12 vs. 58 ± 2.3 cm), a difference that was statistically significant (reproductive state × population type ANOVA *F*_1,91_ = 6.31, *p* = 0.01; effect of limb length *p* = 0.68; Figure 2d).

### 3.2 Fitness components

#### 3.2.1 Fecundity

**Figure 3:**
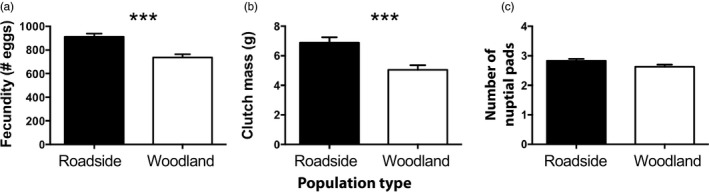
Components of reproductive success were larger in roadside pools. Specifically, female frogs from roadside pools laid larger clutches both in terms of the total number of eggs laid (a) and in terms of the total clutch mass (b) compared to frogs from woodland pools. Male frogs from roadside pools had larger nuptial pads compared to males from woodland pools, although this difference was not significant (*p* = 0.06). Bars show least square mean values (±*SE*) after accounting for variation due to body mass (panels a and b) or forearm length (c). ***p < 0.001

Females from roadside pools laid more eggs (911 ± 27.88 eggs) than did woodland females (737 ± 26.89 eggs), even after accounting for variation due to body mass (ANOVA *F*_1,92_ = 17.48, *p* < 0.0001, effect of body mass *r* ^2^ = 0.31, *p* < 0.0001; Figure 3a). Clutch mass was also positively correlated with female body mass (*r* ^2^ = 0.82, *p* < 0.0001), and roadside females produced 36% more massive clutches (6.89 ± 0.36 g) than did woodland females (5.05 ± 0.31 g; ANOVA *F*_1,69_ = 5.01, *p* = 0.03; Figure 3b).

#### 3.2.2 Larval survival

**Figure 4:**
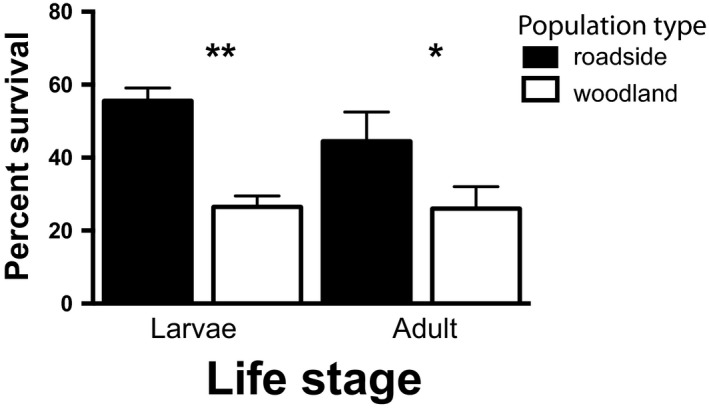
Viability, another component of fitness, was higher in roadside pools compared with woodland pools. Irrespective of their pool of origin, larval survival measured in reciprocal transplant experiments was nearly twice as high for individuals raised in roadside pools compared to woodland pools. Similarly, adult frogs captured at roadside locations had higher survival in terrestrial woodland enclosure experiments compared to frogs from woodland populations. Bars show mean values (±*SE*)

At the end of our larval survival experiment, we found that survival was higher in roadside pools (52%) compared to woodland pools (27%; generalized linear model with log link function (*χ* ^2^ = 8.95, *p* = 0.003; Figure 4)) and this difference arose irrespective of the pool of origin (deme of origin × transplant site: generalized linear model *χ* ^2^ = 0.02, *p* = 0.87).

#### 3.2.3 Adult survival

At the end of our adult selection experiment, we recaptured 19 and 18 adult frogs from the two terrestrial enclosures, respectively (i.e., approximately 37% survival). There was no difference in the survival of males versus females in either enclosure (generalized linear model *χ* ^2 ^= 1.79, *p* = 0.18). However, adult frogs from roadside populations had significantly higher survival (55% and 44%) compared to adult frogs from woodland populations (28% and 24%) in the two enclosures, respectively. Although neither replicate was statistically significant alone, when we pooled data from both enclosures, roadside frogs had significantly higher survival (*χ* ^2^ = 4.51, *p* = 0.03; Figure 4) compared to woodland frogs.

#### 3.2.4 Age distribution

**Figure 5:**
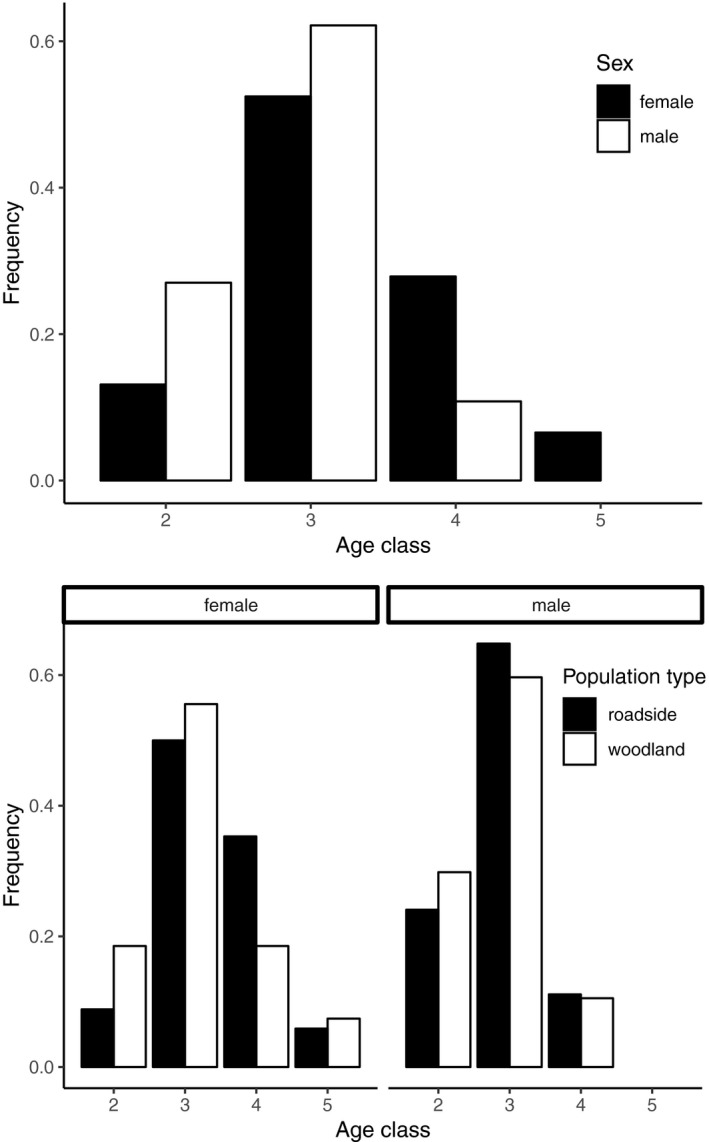
Age class distribution of wood frogs in roadside and woodland populations. Age distribution differed between sexes (top panel) but not between population types (bottom panel: females “f” shown on left, males “m” shown on right). Although not statistically significant, roadside populations tended toward higher frequencies of older individuals (especially among females)

The age distribution of frogs differed between sexes (*χ* ^2^ = 18.2, *df *= 3, *p* < 0.001; Figure 5 top panel) but did not differ by population type outright (*χ* ^2^ = 2.6, *df *= 3, *p* = 0.451) nor by population type when considered separately for each sex (females: *χ* ^2 ^= 2.74, *df *= 3, *p* = 0.433; males: *χ* ^2^ = 0.47, *df *= 2, *p* = 0.791; Figure 5 bottom panel).

## 4 DISCUSSION

We have shown that frogs from woodland and roadside populations differ in components of fitness (female fecundity, larval survival, and adult survival) as well as traits that could influence fitness (adult body size, life history traits, and female jumping performance; Lyapkov, Cherdantsev, & Cherdantseva, 2001; Manly, 1985; Mousseau & Roff, 1987; Wade & Kalisz, 1989). Adult frogs from roadside pools were larger, more fecund, and exhibited superior jumping performance. And although nonsignificant, adult frogs from roadside pools tended to be older than frogs from woodland pools, for instance with twice as many females in age classes 4–5 compared to 2–3. We also showed that wood frog larvae (i.e., tadpoles) survived better in roadside pools compared to woodland pools, which contrasts previous evidence of local maladaptation and “deme depression” at this life history stage (Brady, 2013, 2017).

Roadside pools differed from woodland pools in many important ecological attributes, and one possibility is that these results indicate that the benefit of living in warmer pools with higher levels of dissolved oxygen outweigh the costs of living in water with higher conductivity (i.e., salt concentration) and the pollutants typically found in roadside habitat. Specifically, pools with warmer temperatures (e.g., open canopy ponds) increase growth rates in wood frogs and other pool‐breeding amphibians (Skelly, Freidenburg, & Kiesecker, 2002 #5132) and can therefore result in larger size at metamorphosis, which has been shown to correlate with fitness (Semlitsch, Scott, & Pechmann, 1988; Smith, 1987). However, larval survival in woodland pools studied here was notably low (27%) compared to survival in previous transplant experiments elsewhere (70%–86%) (Brady, 2013, 2017). If this result is atypical, transient fitness dynamics—in which woodland populations are adapted to average but not atypical conditions—might give the impression of relative adaptation in roadside populations and maladaptation in woodland populations when the opposite could in fact be more common (Brady et al. manuscript in review; Brady et al. THIS VOLUME). With such dynamics in mind, we cannot rule out the possibility that ecological attributes of roadside pools protect frogs from exposure to some undetected stressor such as a pathogen (Heard et al., 2015). For instance, chytrid fungus and ranavirus are both potent agents of mortality in amphibians (Abrams, Cannatella, Hillis, & Sawyer, 2013). While we lack empirical evidence of either disease having affected our study populations, conceivably, the markedly lower survival in woodland pools (Figure 4) could have been caused by disease and merits further investigation. If a disease agent is present in woodland pools, its absence from roadside environments would represent an avenue for which roadside environments could be, at least to a certain extent, beneficial to populations susceptible to these pathogens. For instance, Stockwell, Clulow, and Mahony (2015) have shown that infection loads of chytrid fungus are lower in habitats and experimental contexts with NaCl concentrations comparable to those found in roadside ponds. Relatedly, Albecker, Brantley, and McCoy (2018) found that tree frogs from brackish coastal habitats had lower gut nematode burdens compared to freshwater inland populations. Along these lines, roadside pools might provide some protection to wood frogs against fungal outbreaks and other disease agents.

Body size is an important factor that influences the survival of amphibians. Most evidence suggests that larger body size increases the probability of survival (Berven, 1990; Semlitsch et al., 1988) and can even benefit offspring survival (Brady & Goedert, 2017). Large body size may deter predatory snakes from consuming wood frogs and should increase the ability to withstand the winter freeze. Wood frogs are aphagic during winter months, and large body size may facilitate energy storage, which is especially important during movement toward breeding pools in early spring (Costanzo & Lee, 1993). Another possible advantage of larger body size is the enhanced fecundity experienced by females. Females from roadside populations produced larger egg masses (Figure 3a) and greater total numbers of eggs (Figure 3b) compared to females from woodland pools. The fecundity advantages of large body size are well‐understood, and combined with the higher larval survival that we experimentally demonstrated in roadside pools, suggesting that frogs living in roadside pools might have higher fitness than their woodland counterparts, at least periodically in this study area. Finally, larger female body size could also explain the relatively larger nuptial pads on male frogs (Figure 3c) from roadside populations. Males use their nuptial pads (swollen first digits on their front hands) to grasp females in amplexus during mating. Males compete vigorously to monopolize access to females prior to egg‐laying (Buzatto, Thyer, Roberts, & Simmons, 2017; Chuang, Bee, & Kam, 2013; Zhang et al., 2014), and larger nuptial pads help a male ensure sole paternity of a clutch. Alternatively, larger body size might be a consequence of osmotic stress induced by road salt, leading to increased water retention.

Possible explanations for the adaptive differences (higher components of fitness and fitness‐related traits) between adult frogs of woodland and roadside pools include phenotypically plastic responses to different pool environments and/or selection leading to local adaptation to roadside conditions (sensu Brady, 2012). Although the spatial scale of our study does not preclude gene flow between populations (Berven & Grudzien, 1990; Newman & Squire, 2001), wood frog studies conducted on a similar spatial scale have previously demonstrated local adaptation in the contexts of microgeographic variation in forest canopy cover and predation pressure (Skelly, 2004; Urban et al., 2017). Moreover, a recent study demonstrated that exposure to road salt led to wood frog larvae that were larger at metamorphosis, but which subsequently experienced reduced juvenile survival (Dananay, Krynak, Krynak, & Benard, 2015). If salt tolerance is a heritable trait in wood frogs (sensu Gomez‐Mestre & Tejedo, 2004), then increased mortality (i.e., natural selection) could lead to local adaptation in roadside populations of wood frogs. Whether these differences are the result of phenotypic plasticity, evolutionary adaptation, or some combination of the two, our evidence for increased relative fitness and fitness‐related traits stands in stark contrast to previous studies of roadside amphibians (Brady, 2013, 2017; Brown & Brown, 2013; Gibbs, 1998; Karraker, Gibbs, & Vonesh, 2008; Marsh et al., 2008; Reeves, Dolph, Zimmer, Tjeerdema, & Trust, 2008; Trombulak & Frissell, 2000) and to the previous negative reports of road effects more generally (Brown & Brown, 2013; Forman, 2000; Glista, DeVault, & DeWoody, 2008; Parris & Schneider, 2009; Trombulak & Frissell, 2000). In the absence of additional data, we remain agnostic about the sources of these differences in fitness‐related traits.

Although larger egg masses imposed locomotor performance constraints on both groups of females in our study, the locomotor cost was four times larger for females from woodland pools compared to roadside pools (Figure 2d). Better jumping performance in roadside females, even when gravid, is a surprising result that merits further investigation. One possibility is that jumping performance is subject to strong selection during road crossings. Escape speed is positively correlated with limb length and body size in other anurans (Zamora‐Camacho, Reguera, Rubiño‐Hispán, & Moreno‐Rueda, 2014). Mortality associated with traffic may be biased toward smaller and younger individuals, and this might also explain the higher numbers of larger and older individuals in roadside locations. If true, then this result may be evidence of local adaptation in response to traffic (Brown & Brown, 2013). Our data do not fully support this conclusion since we detected no difference in locomotor performance of males and we did not measure traffic selection directly. Alternatively, frogs with higher fitness might select more optimal habitats (such as those that are warmer or with higher dissolved oxygen), which in some years might be found in roadside ponds.

Taken together, our results paint a surprising picture of amphibian populations that appear to have higher fitness in human‐altered environments relative to those in unmodified habitats, despite previous evidence of maladaptation elsewhere in its range. In contrast to the general perception that road construction and use have solely negative consequences for natural systems, our results suggest that some responses may be substantially more complex. Certainly, these effects may be transient (Caswell, 2007), or even cyclical, and future studies would benefit from long‐term (i.e., multigenerational) assessments of the impacts of roads that carefully consider both relative and absolute fitness measures. Still, the possibility of adaptive plasticity or local adaption to roads and traffic should make understanding the implications of fragmented landscapes a priority. This is because the scale of local adaptation will interact with gene flow among populations to either facilitate or slow rates of (mal)adaptive differentiation (Fitzpatrick et al. 2019 THIS VOLUME). For example, habitat fragmentation arising from road networks can limit gene flow (deMaynadier & Hunter, 2000; Swenson & Franklin, 2000; Vos & Chardon, 1998) and may alter the likelihood of local adaptation (Johansson, Primmer, & Merila, 2007). Likewise, the possibility that roads confer benefit against periodic stressors such as disease warrants closer attention. Although evolutionary perspectives on the impact of human‐modified habitat have been slow to take hold, particularly in the contexts of roads (Brady & Richardson, 2017), we suggest that studies of evolutionary adaptation in human‐impacted habitats are increasingly important to understanding the generality of our results and more broadly toward understanding (mal)adaptation in conservation contexts (Derry et al. THIS VOLUME).

## CONFLICT OF INTEREST

None declared.

## Data Availability

Data have been archived in Dryad Digital Repository: https://doi.org/10.5061/dryad.2v41qr7.
